# Supplementary figures and images for: TARPON—A Telomere Analysis and Research Pipeline Optimized for Nanopore
Source: PLoS Comput Biol. 2026 Feb 4;22(2):e1013915. doi: 10.1371/journal.pcbi.1013915 (PMC12871981; doi:10.1371/journal.pcbi.1013915)

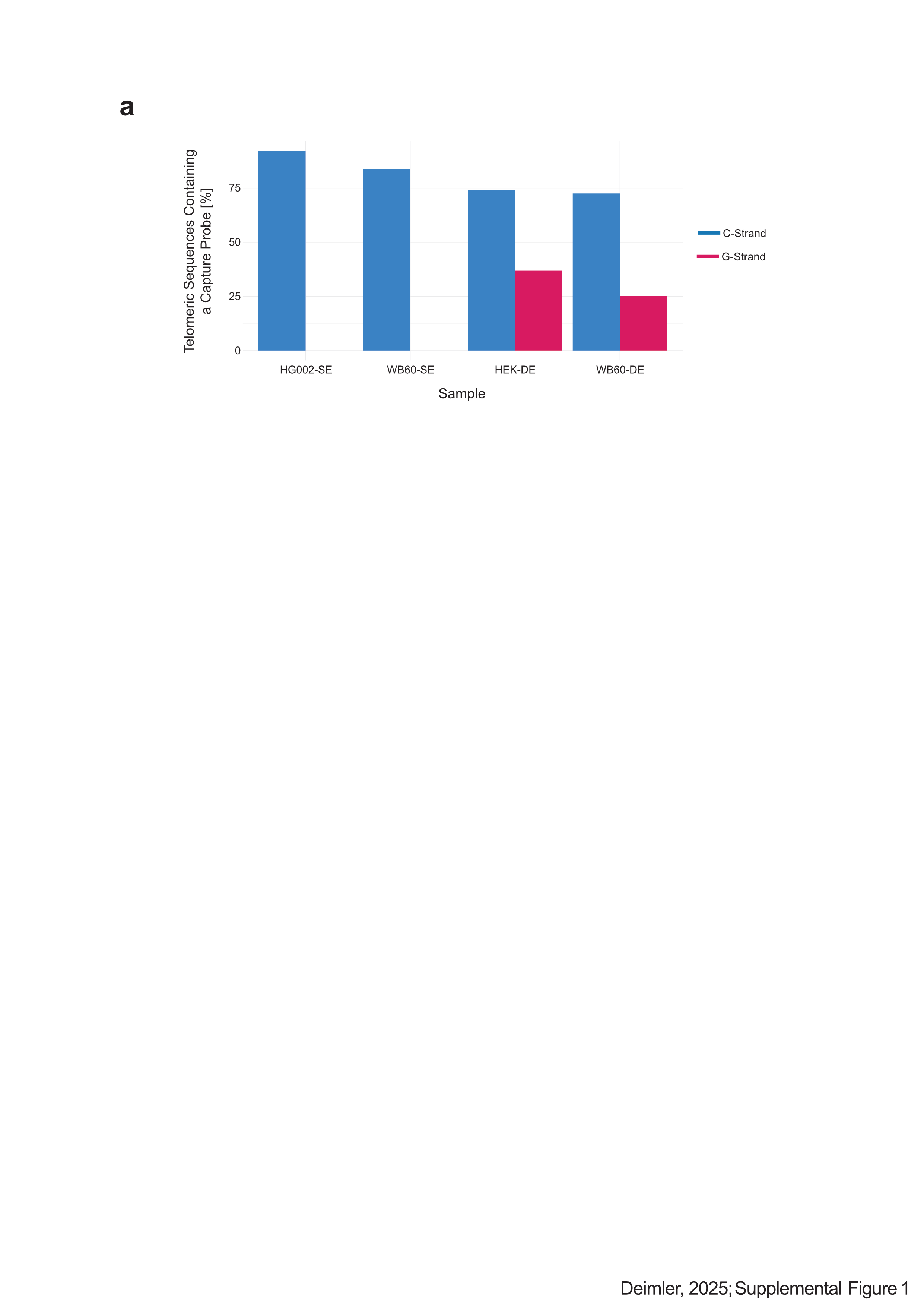

Supplement: S1 Fig — HG002-SE and WB60-SE should not contain G-strand telomeric sequences as the enrichment protocol used should result in only C-strand telomeric sequencing. (TIFF) [file pcbi.1013915.s001.tiff]

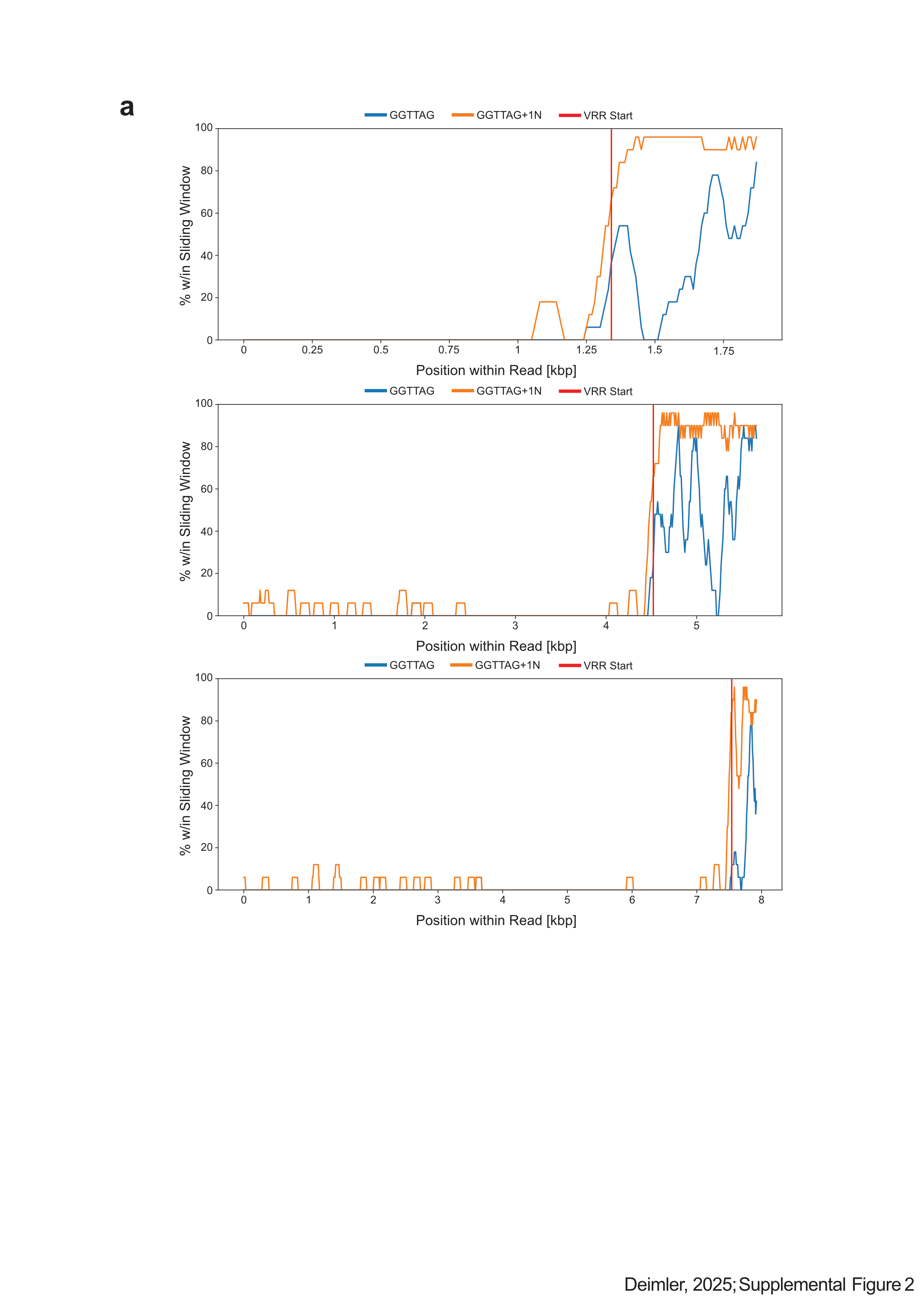

Supplement: S2 Fig — (a) Three examples of reads that end in a capture probe but lack a region of invariant telomeric repeats, instead terminating within the variant repeat-rich regions. Blue lines represent the frequency of GGTTAG repeats within a 100 bp sliding window, orange lines represent the frequency of all telomere + 1N repeats within a 100 bp sliding window, and red lines represent the VRR-region start site. (TIFF) [file pcbi.1013915.s002.tiff]

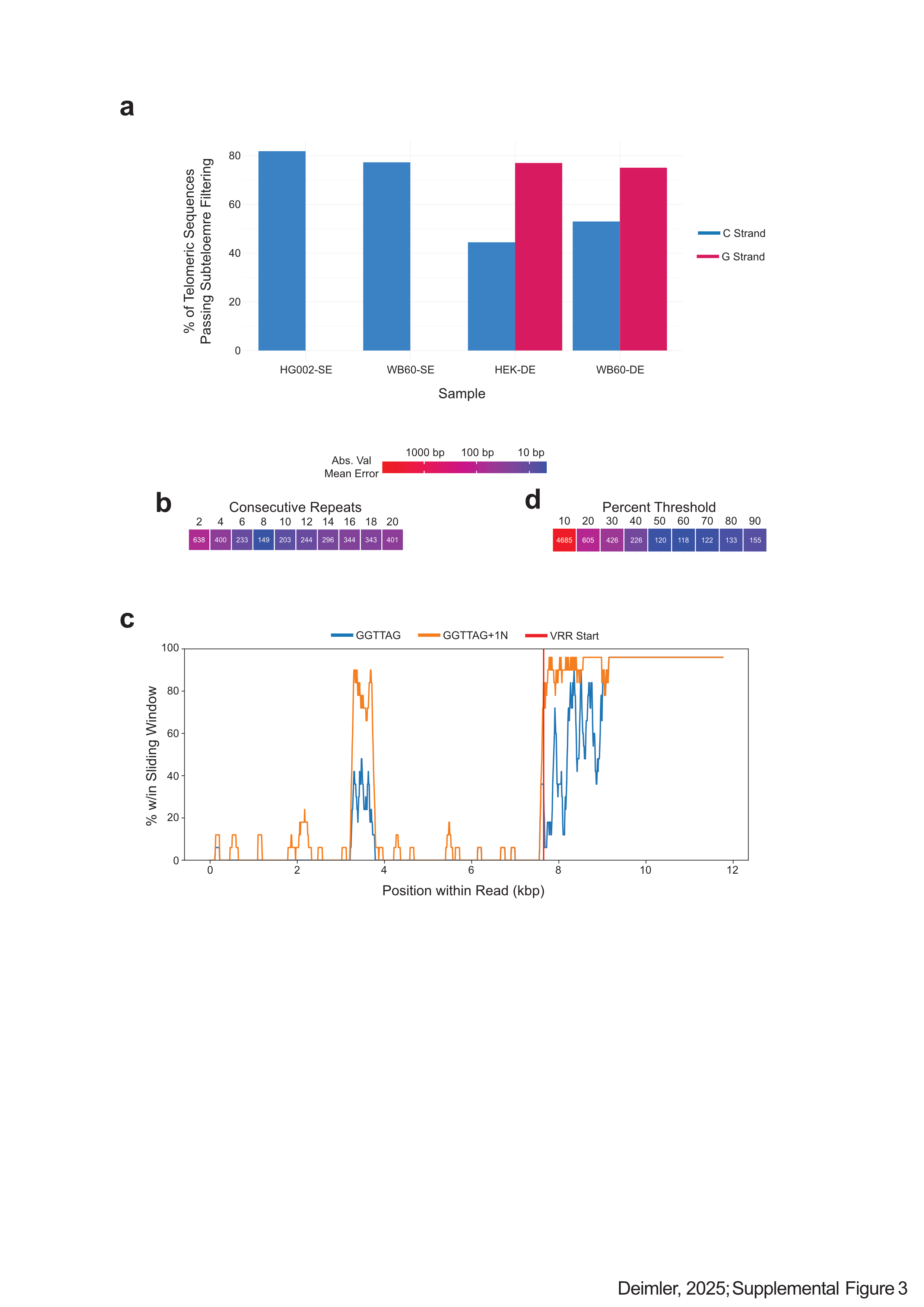

Supplement: S3 Fig — (a) Percentage of telomeric sequences that contain less than 20% telomere + 1N repeats in the first 300 bp of the read opposite of the capture probe separated by strand. (b) Absolute value mean error of the subtelomere to telomere boundary of 400 manually annotated reads defined by a stretch of consecutive telomeric repeats of a given length. (c) An example telomeric sequence that contains a telomere-like island within the subtelomere represented by an increased frequency of telomere + 1N repeats approximately 3.8 kb into the sequence where the blue line represents the frequency of wild type telomeric repeats in a 100 bp sliding window and the orange line represents the frequency of telomere + 1N repeats in the same window. (d) Absolute value mean error of the subtelomere to telomere boundary of 400 manually annotated reads defined by the first sliding window to be composed of greater than a given percentage of telomere + 1N repeats. (TIFF) [file pcbi.1013915.s003.tiff]

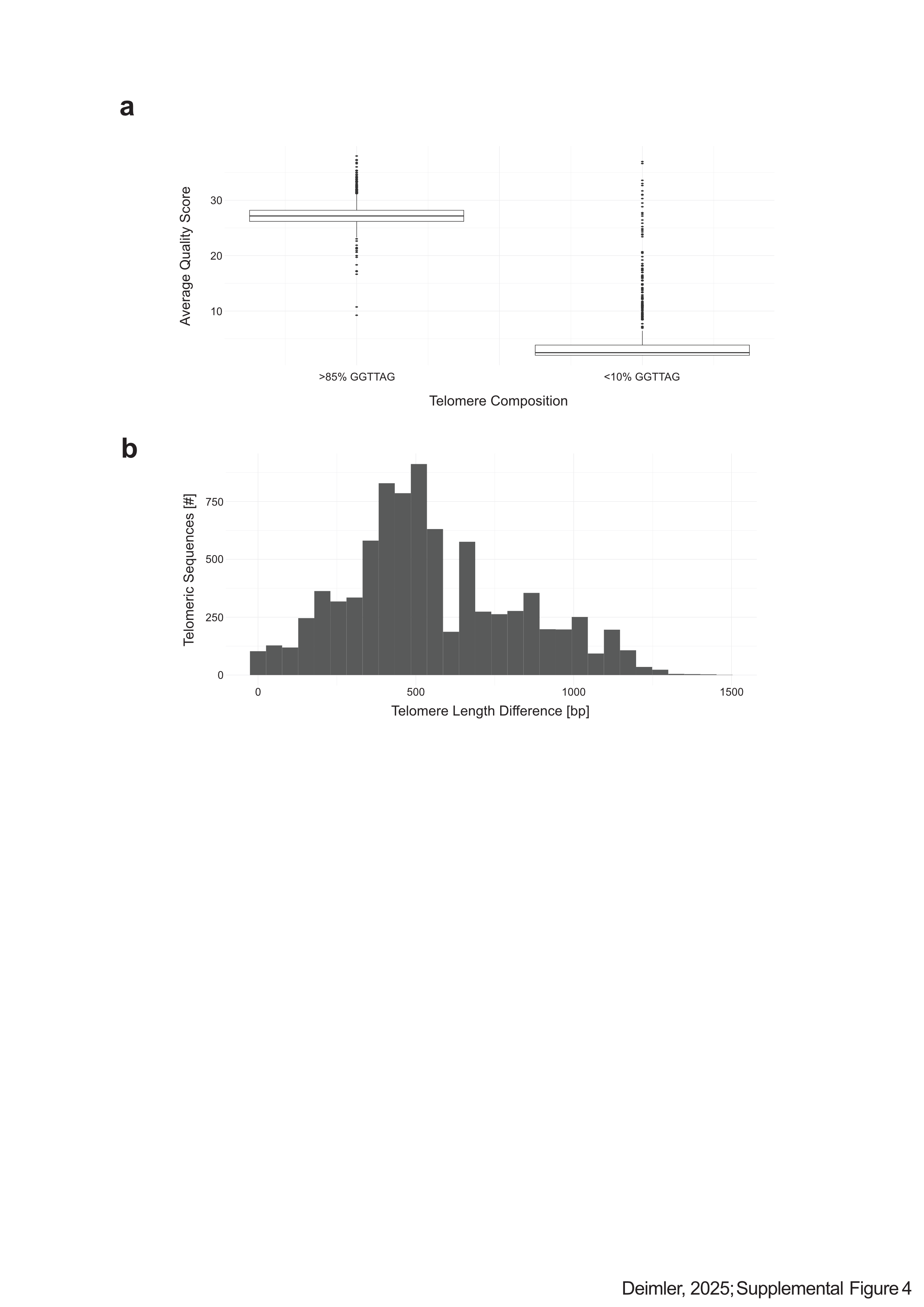

Supplement: S4 Fig — (a) Disitrbution of the average quality score in 100 bp segments of telomeric sequences that are composed of greater than 85% telomere + 1N repeats (real telomeric sequences) and sequences that are composed of less than 10% telomere + 1N repeats after the start of the telomere is identified. Sequences composed of less than 10% telomere + 1N repeats are resultant of basecalling artifacts as seen in Fig 5j. These reads are ultimately removed from analysis by TARPON. (b) The difference between calculating telomere length from the subtelomere-to-telomere boundary to the end of the read compared to the number of nucleotides consisting of wild type telomeric repeats within the same region for all HG002-SE telomeric reads passing all filtering criteria. (TIFF) [file pcbi.1013915.s004.tiff]

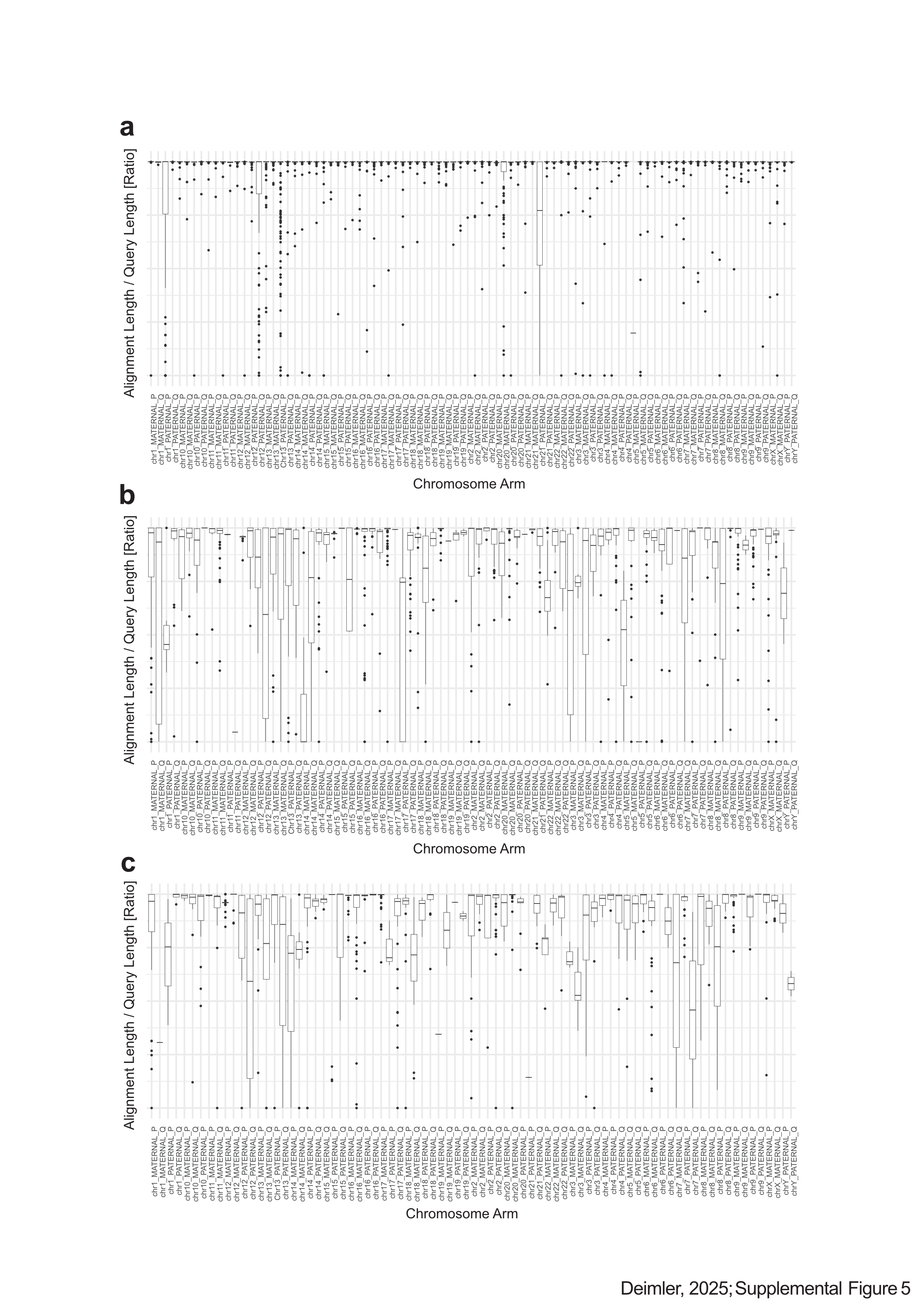

Supplement: S5 Fig — (TIFF) [file pcbi.1013915.s005.tiff]

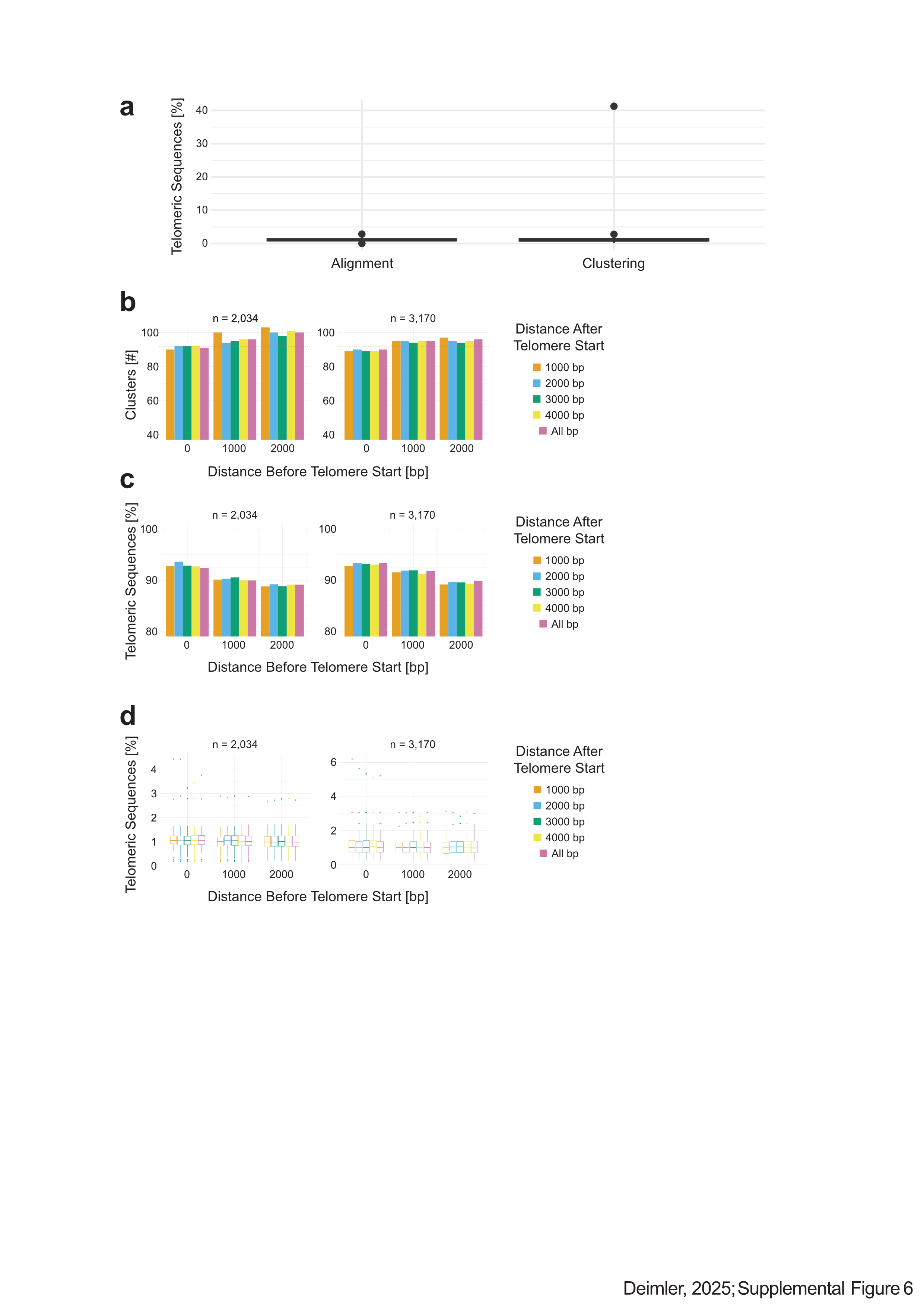

Supplement: S6 Fig — (a) Percentage of telomeres aligning back to each chromosome arm or present in each cluster when full-length telomeric sequences are passed to Telogator2. (b) The number of clusters when non-HG002 samples are clustered using Telogator2 and (c) the percentage of telomeric reads composing said clusters. (d) The distribution of telomeres across all clusters for non-HG002 samples. (TIFF) [file pcbi.1013915.s006.tiff]

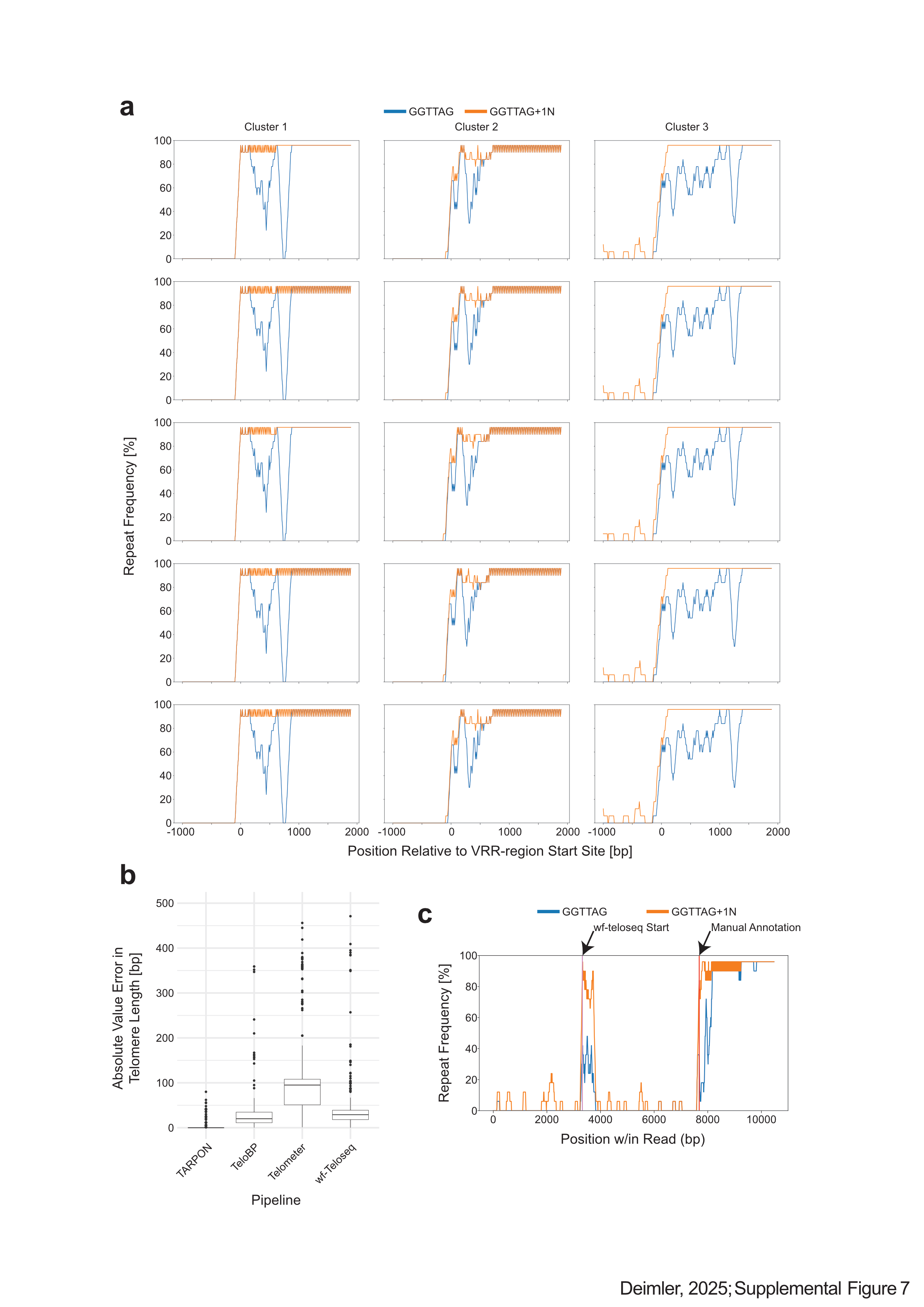

Supplement: S7 Fig — (a) Five example reads from three randomly chosen clusters showing the variant repeat-rich region pattern is identical in a cluster specific manner. Blue lines represent the frequency of GGTTAG repeats and orange lines represent the frequency of telomere + 1N repeats in a 100 bp sliding window. (b) A comparison between the four described telomere analysis software in the accuracy of telomere length prediction compared to the manual annotation of 400 telomeric sequences. (c) The behavior of wf-teloseq in the presence of a subtelomeric island that results in the edge of the island being identified as the telomere start site. (TIFF) [file pcbi.1013915.s007.tiff]

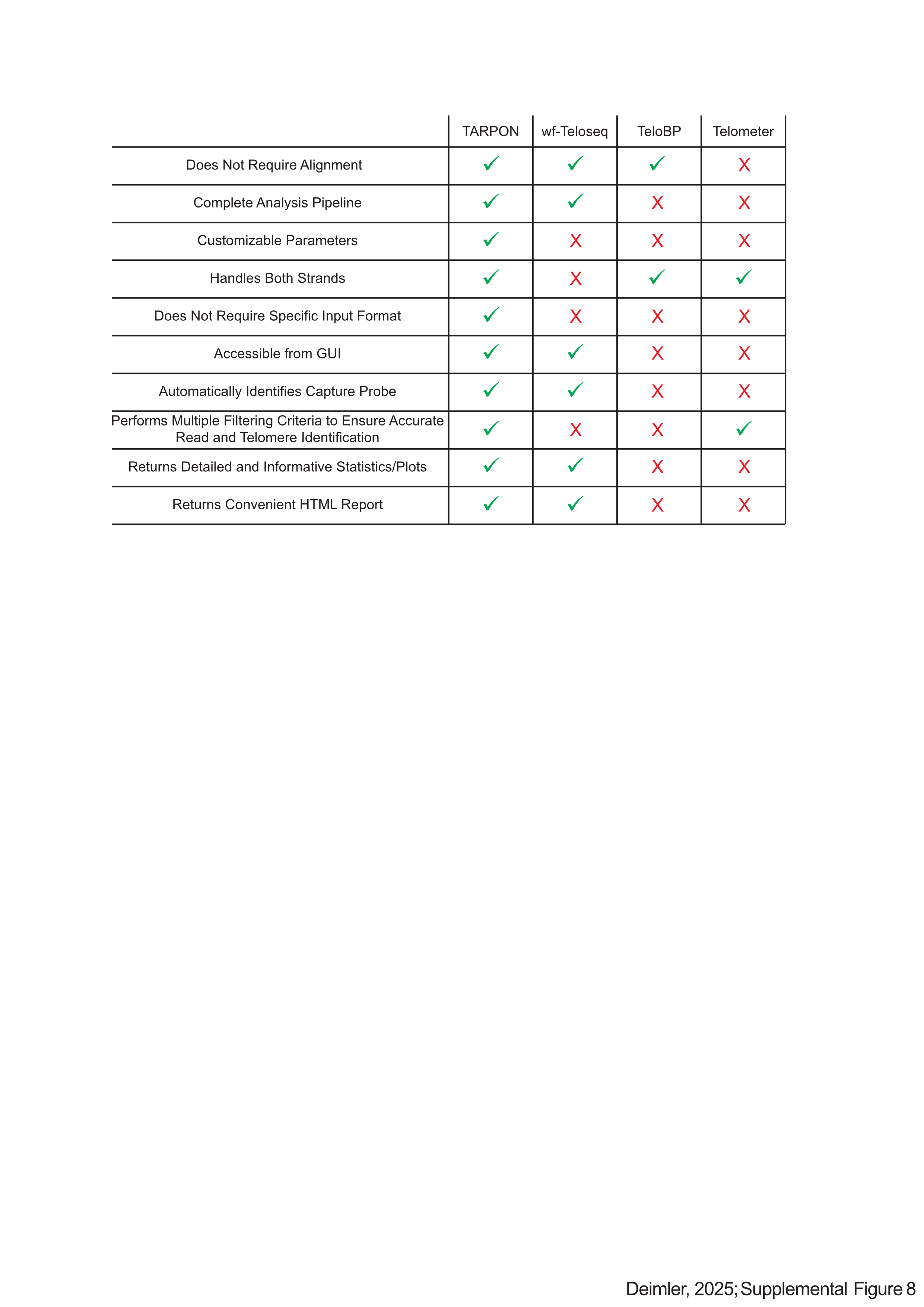

Supplement: S8 Fig — (TIFF) [file pcbi.1013915.s008.tiff]
