# Supplementary material for: TARPON—A Telomere Analysis and Research Pipeline Optimized for Nanopore
Source: PLoS Comput Biol. 2026 Feb 4;22(2):e1013915. doi: 10.1371/journal.pcbi.1013915 (PMC12871981; doi:10.1371/journal.pcbi.1013915)
Supplement: S1 Methods — (a) A description of the samples presented in this study and where appropriate the DNA extraction techniques used. (b) The splint-based telomere enrichment strategy employed in this study for samples HG002-SE and WB60-SE. (c) The duplex-based telomere enrichment strategy employed in this study for samples HEK293-DE and WB60-DE. (d) Relevant parameters to the basecalling of raw Nanopore sequencing data and the execution of TARPON. (DOCX) [file pcbi.1013915.s010.docx]

**Supplemental Methods**

*(a) Sample Selection and DNA Isolation*

Genomic DNA for the below described telomere enrichment protocols had multiple sources. HG002 genomic DNA was purchased directly from the Coriell Institute for Medical Research (NA24385); this sample originates from an immortalized lymphoblastoid cell line and was chosen for analysis because of its publicly available, high quality subtelomeric reference genome. Clinical sample genomic DNA (WB60) originates from whole blood of a 60-year-old individual and was extracted using Chemagen Chemagic DNA Blood Kit Special for 5 mL on the Chemagen Robot MSM1 following manufacturer’s instruction. HEK293T genomic DNA was isolated using the Qiagen Blood and Cell Culture DNA Midi Kit (Cat. no. 13343) following manufacturer’s recommendations.

*(b) Splint Capture Based Telomere Enrichment*

Telomeric sequencing via the ONT Telo-Seq protocol was conducted following manufacturer’s recommendations. Briefly, 15 µg of genomic DNA was incubated with Telo Adapter mix (TLO, 100 nM) in 200 µL reactions containing 1X rCutSmart Buffer, 1 mM rATP, and 50 U/µL Quick T4 DNA Ligase (NEB36056) at 35°C for 16 hours, followed by heat inactivation at 65°C for 10 minutes. The ligation reaction was split into four 50 µL aliquots. EcoRV-HF (20 U, NEB R3195S) was added to each aliquot and incubated at 37°C for 30 minutes to digest bulk genomic DNA, followed by heat inactivation at 65°C for 20 minutes. The aliquots were pooled and subjected to 3’ dA tailing using NEBNext dA-tailing reaction buffer and 45 U Klenow Exo- (NEB E6053) in a 250 µL reaction at 37°C for 30 minutes.

SPRI bead purification (1x) was performed using AMPure XP beads (Beckman Coulter A63882). Beads were pelleted on a magnet and washed twice with 80% ethanol. DNA was eluted at 37°C for 15 minutes with 198 µL nuclease-free water. DNA (196 µL) was incubated in a 200 µL reaction containing 100 nM Telo-Splint (TLS; 2 µL of 10 µM stock), 50 mM NaCl (2 µL of 5 M), and held at 50°C for 1 hour. The Telo-Splint contains a 5-nt overhang complementary to the ONT native adapter. The Telo-Splint/TLO combination is hereafter referred to as the "capture probe" to differentiate it from the ONT sequencing adapter.

A 0.5X SPRI bead purification was performed as above, and DNA was eluted into 32 µL nuclease-free water. Sequencing adapter ligation was performed using 10,000 U Quick T4 DNA Ligase (NEB E6056) and 5 µL native adapter in 1X NEBNext Quick Ligation buffer at 21°C for 20 minutes. Another 0.5X SPRI bead cleanup was carried out, followed by two washes with long fragment buffer (LFB). DNA was eluted into 25 µL elution buffer and loaded onto a R10.4.1 MinION flow cell for a 72-hour sequencing run.

*(c) Duplex Capture Based Telomere Enrichment*

Telomeric sequencing via a duplex capture approach was conducted using a modified version of Karimian et al. (2024). A biotinylated oligo (Oligo BLoli9407, Supplemental Table 1) was annealed to a complementary oligo with a three-telomeric-repeat overhang (Oligo BLoli9406, Supplemental Table 1) in 1X Annealing Buffer [20 mM Tris/Ac (pH 7.5), 50 mM NaCl, 2 mM MgCl_2_] at 20 nM final concentration. The mix was heated to 99°C for 1 minute and cooled to room temperature at 1°C/min.

This duplex, hereafter referred to as the "capture probe," was ligated to 3 µg genomic DNA in 50 µL reactions (16 replicates) containing 75 fmol capture probe, 1X HiFi Taq Ligase Buffer (B0647SVIAL), and 1 µL HiFi Taq DNA Ligase (M0647SVIAL) using a MasterCycler X50s. Ligations ran for 5 minutes at 65°C, followed by 25 cycles of 1 minute at 65°C and 3 minutes at 45°C with 1°C/s ramp rate. EcoRV-HF (20 U) was added and incubated at 37°C for 2 hours.

MyOne C1 streptavidin beads (16 µL) were washed and resuspended in 816 µL 2X B&W buffer [10 mM Tris-HCl (pH 7.5), 1 mM EDTA, 2 M NaCl]. Digestion reactions were pooled, combined with beads, and rotated at room temperature for 45 minutes. Beads were collected on a magnet, the supernatant discarded, and the beads washed twice with 300 µL 2X B&W, once with 300 µL Elution Buffer [10 mM Tris-HCl, pH 8.5], followed by resuspension in 72 µL 1X rCutSmart Buffer.

PvuI-HF (3 µL, 60 U, NEB R3150L) was added and incubated at 37°C for 90 minutes with rotation. The reaction was heated to 65°C for 20 minutes to release digested DNA, which was retained after pelleting the beads. Library preparation was completed using the ONT LSK114 kit (GDE_9161_v114_revX_13Dec2024) following the manufacturer’s protocol.

*(d) Basecalling and TARPON Pipeline Execution*

Pod5 files were basecalled using Dorado v0.7.0 with Fast, High Accuracy (HAC), and Super Accuracy (SUP) v5.0.0 models on NVIDIA L4 GPUs. To reduce compute time, telomeric reads were first identified in fast basecalled data using TARPON and re-basecalled with SUP if --pod5_directory and --fast_basecalled flags were specified. TARPON is implemented in Nextflow v24.04.3 with Docker containerization. Input data may be pre-basecalled with fast or SUP basecalling models; no preprocessing is required. TARPON detects telomeric reads based on the presence of a user-defined telomeric repeat (default: GGTTAG).

Strand-specific filtering is applied via the --c_strand_only flag for splint-enriched libraries. Filtering thresholds, barcode demultiplexing, capture probe sequence, and GUI/CLI options are detailed in the GitHub README. The instructions on how to run this pipeline from the command line or the EPI2ME GUI and the pipeline’s source code are available at <https://github.com/baumannlab/TARPON>.
