# Supplementary material for: TARPON—A Telomere Analysis and Research Pipeline Optimized for Nanopore
Source: PLoS Comput Biol. 2026 Feb 4;22(2):e1013915. doi: 10.1371/journal.pcbi.1013915 (PMC12871981; doi:10.1371/journal.pcbi.1013915)
Supplement: Attachment — Submitted filename: Response_to_Reviewers.docx [file pcbi.1013915.s012.docx]

We thank the three reviewers for their thoughtful and constructive comments. The changes to the text and figures made in response to each question raised have made this manuscript clearer and stronger. Below we have responded to each comment in blue.

**Reviewers' comments:**

Reviewer's Responses to Questions

**Comments to the Authors:
Please note here if the review is uploaded as an attachment.**

Reviewer #1: Deimler et al. describe a software pipeline (TARPON- Telomere Analysis and Research Pipeline Optimized for Nanopore) for computationally detecting and analyzing telomere sequence-containing DNA reads derived from Oxford Nanopore Technology (ONT) single-molecule long-read datasets. The pipeline has several important general advantages over other existing pipelines for analyzing telomere-containing nanopore reads, including ease of use combined with easily modifiable parameter modifications by non-bioinformaticists; modification of key analysis parameters through either GUI or command line interfaces and integration of all steps into a single Nextflow pipeline ensures reproducibility and ease of use. In addition, several important and useful innovations are incorporated into their pipeline; (1) a broader definition and systematic incorporation of (TTAGGG)n-like repeat(s) (which they refer to as GGATTG +1N) into subtelomere-telomere boundary definition, with their consequent incorporation into all downstream telomere terminal repeat analysis (telomere length determination, and potential (TTAGGG)n -like repeat composition and organization analyses); (2) automated detection and analysis of terminal telomere repeat tracts, telomere capture probes, and sequence tags used for multiplexing samples and (3) a set of thoughtfully designed and empirically tested filtering steps to help ensure that only reads with full-length terminal repeat regions are included for telomere length determination and other downstream analyses.

The logic, clarity, and detail for the results underlying Figures 1-5 (pre-mapping telomere read detection, filtering steps, and telomere length & composition analyses of telomere-containing single-reads) is for the most part commendable. Exceptions to this which require remediation are:

We thank the reviewer for their positive assessment and summary of TARPON while also providing insightful suggestions for improvement. We have clarified and addressed open questions the reviewer has identified.

(A) Fig 1C, which has print content much smaller (about 10-fold ?) than the rest of the figures and is nearly impossible to read.

Fig 1b and 1c were removed from the manuscript. Unfortunately, as Epi2Me is a licensed software we are unable to include screenshots of the program due to copyright law.

(B) The precise definition of GGATTG + 1N, which is ambiguous as written. Are 5-mer, 7-mer, and 6-mer single-base substitution variants included ? Does this set of variants comprehensively cover the known human (TTAGGG)n -like variants in terminal repeat tracts ? There should be sufficient high-quality telomere sequence data publicly available to actually check this, some effort should have been made to determine whether this variant set actually covers known variants, or if some are missed. A supplementary table/comprehensive list showing all variants used for this pipeline should be provided.

Telomere+1N repeats refers to wild type telomeric repeats and all one nucleotide substitutions of the wild type telomeric repeat sequence excluding one nucleotide insertions or deletions. This has now been clarified in the text (lines 311-313, “We refer to this combined pattern of canonical repeats and single-nucleotide substitution repeats as "telomere+1N" and does include insertions or deletion of the wild type repeat.”). When running Illumina short read WGS datasets of clinical samples through Computel, more than 98% of the repeats fall within the definition of telomere+1N. We solely use this definition of a universe of variant repeats to consistently and reproducibly identify the telomere to subtelomere boundaries on each chromosome arm. Exclusion of a small subset of repeat variants containing insertions, deletions or two or more nucleotide substitutions from the universe of what we consider telomere repeat variants does not impact the boundary positions. We agree however, that repeat sequence variations constitute a very interesting biological phenomenon, and we hope that readers will utilize nanopore sequencing and TARPON analysis tools to better characterize the subtelomeric regions and VRR-region in a wide range of samples.

(C) Several overly sparse figure legends, and confusing figures which do not clearly describe the data presented. For example:
4b is unclear, plotting % repeats in the “first 300 bp of a read” for the four datasets – “first” from which end of the read and for which strand ? Please clarify in the figure legend. This criterion caused removal of lots of reads from the datasets (4c), so its critical to understand whats happening and to do this correctly.

Figure legends have been revised to provide additional detail. Specifically for Figure 4b, TARPON reverse complements C strand reads into the G-strand orientation (lines 226-227, “Reads classified as C-strand telomeric reads (<20% G-strand content) are reverse complemented into G-strand orientation for downstream analysis.”). Following this step, every explanation refers to the G-strand orientation. Strand specificity is tracked as an additional tag in the BAM file and is returned in the final stats report. The text and figure legends have been updated for further clarification of where this region lies within the analyzed reads (lines 323-333, “However, while plotting the percentage of telomere+1N repeats within the first 300 bp of a read (the subtelomere end of the read), a notable bimodal distribution emerged: most reads contained less than 15% telomere+1N repeats, while a smaller subset exceeded 80% (Fig. 4b). This latter group represents telomere-containing sequences that begin within the VRR-region or even within homogenous GGTTAG repeat sequence. These reads were excluded from further analysis as proximally truncated, since the full length of the VRR-region or telomere sequence cannot be determined with confidence (for C-strand reads, this uncertainty applies to the read end since all reads have been reverse complemented into the G-strand orientation).”; Figure 4b figure legend, “The percentage of telomere+1N repeats in the first 300 bp of a read in the subtelomere direction with a 20% threshold indicated by a dotted red line.”). Additional information has been added to the legends of Figure 4-6 as well as Supplemental Figures 1-7.

4d-4h: The steps to define the subtelomere-telomere boundary in 4d-h should be moved to supplementary figures and described in detail there, including expanded figure legends and relevant text from the main paper. Only the final best conditions should be shown in the main part of the paper, since these are the ones defined for use; summary figures 4i and 4j should also be included in the main part of the paper here.

Tests to identify the subtelomere to telomere boundary that were not used in the final pipeline have been moved to the supplement including panels 4d and 4e. Panels 4 f, g, and h remain in the main text as these show crucial parameters in identifying the subtelomere to telomere boundary that the user can redefine if desired.

Fig 5a X-axis states “Single Nucleotide Repeat Composition [%]”. What does this mean ?

This has been updated to Telomere+1N Repeat Composition [%].

Fig 5g – What do the colors designate ?

The colors represent the four sequencing runs performed as shown in Figure 5h. The colors had no significance in Fig 5g/k and have therefore been removed.

Supplemental Figure 5 – Please explain in more detail what this is and how it was determined.

The figure legend as well as the main text were updated to include more details; (other changes have resulted in this now being supplemental fig. 4). However, the underlying basecalling artifact is inherent to the nanopore dorado basecaller. ONT is aware of this issue and after having multiple conversations with their programmers, they have not been able to identify the root cause. In certain sequencing runs, telomeric sequences produce erroneous stretches of base calls. If basecalling is repeated, the same reads exhibit this artifact in the same locations. This is described in lines 386-392 in the manuscript. An example read in question is presented in Fig 5j. Such reads are excluded from further analysis. (Figure Legend 4a, “(a) Average quality score in 100 bp segments of telomeric sequences that are composed of greater than 85% telomere+1N repeats (real telomeric sequences) and sequences that are composed of less than 10% telomere+1N repeats after the start of the telomere is identified. Sequences composed of less than 10% telomere+1N repeats are resultant of basecalling artifacts as seen in Fig. 5j. These reads are ultimately removed from analysis by TARPON.”; lines 386-392, “Reads resembling Fig. 5j, which exhibit a sharp transition from fully telomeric sequence to 0% telomere+1N content, are attributed to sequencing artifacts introduced by dorado v0.7.0. These segments, composed of less than 10% telomere+1N repeats, typically show reduced quality scores relative to the rest of the telomeric sequence, composed of greater than 85% telomere+1N repeats, (Supplemental Fig. 4a) and lack consistent VRR-region repeat patterns indicating this is not a chromosome arm specific or biological phenomena.”)

(D) The actual capture sequences used for the Splint-capture libraries comprising two of the source datasets analyzed in the paper were not provided. This makes it difficult to precisely replicate the entire pipeline.

Supplemental table 1 has now been updated to include the splint enrichment telo-splint sequence.

A major issue is that the section of the paper describing the mapping and clustering of telomere reads (the results shown in Figure 6 and associated supplementary figures) does not provide sufficient detail to understand what was done and what the results are supposed to be showing. This single-telomere-read mapping and telomere read clustering section is fraught with major issues and uncertainties that are not addressed adequately.

Specifically:

E)
How exactly was the read-mapping done ? There is no methods section describing the exact algorithms and parameters used to acquire the results shown. I’m assuming some version of minimap2 was used, but insufficient discussion of the mapping parameter threshholds used for declaring a read “uniquely mapped” is given. For example, how is the Mapping Quality Score arrived at ?

The manuscript text has been updated to better describe the mapping methodology (lines 420-426). In Figure 6a, various mapping qualities are tested to determine the number of uniquely mapping or multimapping reads. This has now been further clarified in the text: “Alignment was performed using minimap2 v2.26-r1175 and default parameters with the mapping option “-ax map-ont” specified. Alignments were then filtered for a minimum mapping quality (MAPQ) of 0-10, 20, 40, and 60 prior to determining the number of uniquely mapping reads (reads that have only one alignment with a MAPQ equal to or greater than the specified quality score), unmapped reads (reads with no alignments), and multimapping reads (reads with multiple alignments with a MAPQ equal to or greater than the specified quality score).”

Will this vary with the basecaller, does it mean the same thing near telomeres as it does in less complex genome regions, and what are the variables contributing to this number ? These parameters as well as the actual nanopore read quality within subtelomere regions are expected to be critical for assessing the level of certainty that a read mapping is unique and correct. Especially for the relatively short distal stretches of subtelomeres that seemed to be the main focus of the read mapping and clustering analyses, there are highly similar hypervariable VNTRs as well as variable organizations of highly similar segmental duplication segments at many subtelomeres.

The quality and sequence accuracy of reads will vary depending on the basecaller used, with SUP providing the most accurate read sequences. We therefore recommend performing read clustering/alignment in chromosome arm specific manners with SUP basecalled data. This was previously only mentioned in the software’s README file but has now been added to the manuscript’s text as well (lines 416-419, “Accurate assignment of each telomeric read to a unique chromosome arm is therefore an important goal and only high quality, SUP basecalled data should be used to identify chromosome arm specificity.”)

While the mapping process is treated as a black box, the data presentation seems to emphasize the positives. For example, while Figs 6a – 6c indeed suggest (as expected) that the telomere read mapping works best using a reference sequence source genome identical to that from which the telomere reads were derived, it doesn’t really address how accurate the single-telomere mapping specificity is for these 1-pass error-prone reads. The argument for telomere specificity of read-mappings is made in part by showing similar telomere read coverages at all chromosome ends in HG002 (6b); but it seems to be made after averaging the number of reads at all arms contributing to the lowest quartile of reads per arm, and using that number to normalize the mapping number per chromosome arm. Why not instead provide chromosome end by chromosome end raw read mapping numbers here, and let the reader dig in to these mapping data to investigate individual reads ? In Figure 6c, what is the average query alignment length as a fraction of the length of the subtelomeric part of the telomere-containing read (are there significantly sized subtelomeric segments of query reads not aligning or mis-aligning to the reference ? – and if so, how does this vary by chromosome end ?).

Figure 6b has now been updated to include the percentage of telomeric sequences aligning back to each chromosome arm to depict the presented issue with aligning telomeric reads to a non-sample specific subtelomere reference – the values provided in the text have been appropriately updated on lines 439-452. Additionally, a novel panel has been added to figure 6 (panel d) to show the average length of the subtelomeric portion of the query alignment as a fraction of the subtelomeric length of the telomere containing read and three supplemental panels have been added to supplemental figure 5(a-c) to demonstrate this in a chromosome arm specific manner across the three samples in question when aligning to the HG002 subtelomeric reference. “The poor alignment quality can additionally be seen when comparing the subtelomeric alignment length against the subtelomeric length of the query sequence (Fig. 6d). While this ratio is near 1 for HG002 telomeres aligned to the HG002 reference, this decreases substantially for other reference genomes or clinical samples. Interestingly, while few differences exist in a chromosome arm specific manner in the alignment of HG002 sequences (Supplemental Fig. 5a), certain chromosome arms in clinical samples (Supplemental Fig. 5b and 5c) exhibit more complete subtelomeric alignments than others. It remains to be seen if this is simply an artifact of alignment or a global conservation in certain subtelomeric structures.”

F) There are similar issues with the clustering of telomere reads by sequence similarity using Telogator2 (very little description of what Telogator2 is and how it works, another “black box” producing clusters of unknown quality/confidence from the nanopore telomere reads).

Telogator2, as used by the authors, is a previously published pipeline for the clustering of telomeric sequences (Stephens and Kocher, 2024). While the initial publication does not describe the pipeline in great depth, the study has passed peer review and the code is publicly available. It is beyond the scope of this paper to further describe the clustering algorithm published by others. Instead, we benchmark its ability to accurately cluster reads. “Telogator2 (commit #d4e50d1) (31,38,39), a pipeline designed to derive chromosome arm-specific telomeric length from long reads, can be used to cluster telomeric reads without alignment to a reference using pairwise alignment and hierarchical clustering. Perfect clustering would result in the formation of 92 clusters with 100% of the input telomeric sequences divided evenly across the clusters (~1.08% of the sequences per cluster).“ An additional sentence was added with the exact parameters used while running telogator2 to ensure the clustering performed in this manuscript is not a black box: “When full length telomeric sequences were used as input for Telogator2 (-tt 0.1 --collapse-hom 500 -r ont -p 10 --filt-tel 0 --filt-nontel 10000 --filt-sub 0 –debug-noanchor) with parameters designed to turn off read filtering “

From previously published work, Telogator2 seems very effective for clustering of telomeres using (TTAGGG)n-like repeat patterns in the proximal region of terminal repeat tracts sequenced with high-quality HiFi methods, but it worked poorly with nanopore reads base-called several years ago and it remains unclear how effectively it might work with enriched telomere libraries sequenced using current nanopore methods and current basecallers. As with some of the other results, Figures 6d-g describing the clustering results were difficult to follow because of very sparse figure legends and confusing figure labels (eg., distance before telomere start, distance after telomere start, telomeric sequence %, telomere sequence #, telomeric sequence per cluster %, clusters #). No rationale is given for why telomere clusters are defined as containing >0.02% of the total number of input telomere reads. There is no descriptor or metric that I could ascertain amongst the results for measuring cluster quality, which would seem to me to be an extremely important parameter. It seems like HG002 might be an ideal model to develop Telogator quality metrics, as HG002 assembly used HiFi sequences extending into telomeres, and the current study includes telomere-enriched libraries sequenced from HG002 using current nanopore methods. As currently written, I cannot understand and don’t really trust the clustering results in Figure 6.

Telogator2, in its version that was used in this manuscript, was ineffective at clustering telomeric sequences (Fig 6d) when using the entire telomeric read sequenced. We cannot comment on its effectiveness with PacBio long read sequences as we have not analyzed PacBio long read data. However, by trimming the nanopore read to windows of variable size around the variant repeat rich region (specified as distance before telomere start and distance after telomere start), the number of clusters and size of the clusters approached the expected values of 92 clusters that contain approximately 1.08% (100/92) of the telomeric reads each. The manuscript text has been modified to make it clear what region of the telomeric reads was used in clustering. (lines 483-488,“To focus clustering primarily on the VRR-region, telomeric sequences were trimmed to the sequence found immediately before or after the telomere start identified by TARPON. When reads were trimmed to contain 3 or 4 kb of subtelomeric sequence (distance before telomere start) and clustered, similar trends to full length reads are observed with fewer clusters than expected, regardless of the length of telomeric sequence (distance after telomere start) (Fig. 6e). “Additionally, a sentence stating why a minimum cluster size of 0.02% was required has been added (this is simply because telogator2 will return all reads entered into the analysis, regardless of cluster size resulting in many clusters that are comprised of only one or two reads. “A cluster is retained for further analysis only when containing greater than 0.02% of the input telomeric sequences. This removes very small artificial clusters created by Telogator2 composed of as few as one or two telomeric reads.” This is why the percentage of telomeric sequences included into clusters (fig 6e) is less than 100%.

The reviewer is correct that HG002 would be the ideal model to develop telogator2 quality metrics, which is why we compare the clustering of HG002 reads by telogator2 to the alignment of the reads to the HG002 subtelomeric reference in this manuscript. Figure 6h shows that each cluster has a 1 to 1 relationship with a chromosome arm’s mapping results. (lines 501-503 “Furthermore, read IDs belonging to each chromosome arm by alignment are uniquely found in a single cluster (Fig. 6h) demonstrating the accuracy of the clustering methodology.”) When visually inspecting these clusters (Supplemental Figure 7a), each read within a given cluster has an identical VRR-region pattern. “Additionally, visualization of the clusters by plotting individual telomeric sequences and comparing the VRR-region structure further supports the accuracy of the clustering methodology (Supplemental Fig. 7a).” The figure legend of Figure 6 has been rewritten for further clarity: “Figure 6. Chromosome Arm-Specific Telomere Length**.** (a) Number of uniquely mapping reads, multimapping reads, and unmapped reads of 3 different telomeric sequence datasets aligned to 3 different subtelomeric reference genomes. (b) The chromosome arm distribution of telomeric sequences when aligning to different reference genomes with the expected 1.08% of sequences mapping to each chromosome arm annotated with a dotted red line. (c) The percentage of the alignment length that is composed of gaps or mismatches when aligning the three datasets to three different subtelomeric references and (d) the length of the alignment as a ratio to the length of the query sequence. (e) Number of clusters composed of greater than 0.02% of the input telomeric sequences when trimming the telomeric sequences to consistent lengths and (f) the percentage of input telomeric sequences contained within one of the clusters documented in panel e. (g) The distribution of telomeric sequences across the clusters directly compared to the distribution of the same telomeric sequences aligned to the HG002 subtelomeric reference genome (red). (h) The number of clusters that align to each chromosome arm in the HG002 subtelomeric reference in relations to the distance before and after the telomere start site. One indicates that alls read aligning to the same chromosome arm are found within a single cluster.”

Reviewer #2: Paper Summary:

In this paper, the authors have developed a software called TARPON which can perform end-to-end chromosome arm-specific telomeric sequence and length analysis specialized for ONT reads. This is designed as a Nextflow pipeline that can either be executed via the command-line or the EPI2ME GUI. With respect to the existing telomere analysis tools like Telometer, TeloBP, and wf-teloseq, TARPON provides a better all-in-one experience for the users by taking over the data pre-processing steps within its integrated workflow. Unlike pre-existing tools, TARPON can serve as an easy-to-use tool for non-expert users with its default settings and also offers increased flexibility to the advanced users without needing to modify the source code.

Strengths:

- Well-written: The manuscript is easy to read and explains all the components in a comprehensive manner. The authors have included the limitations of the current version of the software. They have also suggested how the users can optimize the parameter values for different application scenarios.

- Meticulous methodology: TARPON’s workflow consists of novel components which are not present in the existing telomere analysis tools. The authors started with details of the lab protocols followed for sample preparation which ensures reproducibility. The authors later explained each step involved in the TARPON pipeline with adequate reasoning.

- Comprehensive experiments and result analysis: The authors performed thorough experimentation on diverse datasets and reported the results with detailed illustrations. The result analyses are sufficient to validate the performance of TARPON on human samples.

- High quality illustrations: The authors have performed thorough experimentations and corresponding analyses. The results are presented using high quality and easily interpretable figures.

- Strong motivation and applicability: The manuscript explains the shortcomings of TRF, qPCR, and FISH. It also explicates the benefits of utilizing ONT reads with enrichment techniques for telomere analysis with respect to cost and efficiency.

- Well-organized outputs: TARPON generates the outputs in publication-ready format including customizable statistics and summaries.

- Easy-to-follow instructions: The software installation instructions are well documented. The users don’t require in-depth computational knowledge to perform them.

We thank reviewer 2 for their detailed analysis and positive assessment of TARPON. Their assessment captures precisely what we hope to provide to the telomere research community: “a better all-in-one experience for the users.” We are pleased to hear that the instructions for installation were easy to follow. In our responses below we have addressed the open questions the reviewer has identified.

Major Questions:

- Although the authors claim that TARPON is applicable to telomere analyses in variant-rich samples and organisms (some insects and plants) with non-canonical telomeric repeats, they don’t provide any experimental validation for this claim. Did the authors run experiments on those datasets, but omitted the results in the manuscript? In that case, it would be interesting to get a look at those results.

We have not run the pipeline on datasets from insects or plants. We designed TARPON to be customizable and accommodate repeat variation through the modification of certain parameters. The manuscript text has been updated (line 761) to explicitly state that this has not yet been tested. “Importantly, telomere analysis with TARPON is not limited to human samples. TARPON can be used to study telomere length dynamics in any organism with non-heterogeneous telomeric repeats, including many invertebrates and plant; however, these uses remain untested.”

- It was intuitive that results obtained from the ONT reads and the corresponding reference from the same sample (HG002 in this case) would be the best. Likewise, when analysis is done between reads and reference coming from different samples would perform poorly. I am not sure what new information the readers would gain from this set of experimental analysis.

We completely agree with the reviewers’ statements. These mis-matched comparison were specifically included in the manuscript following publication of other studies (Karimian et al., 2024; Schmidt et al., 2024) that aligned HG002-unrelated clinical data to the HG002 reference and presented chromosome arm specific telomere length conclusions. Additionally, telometer requires an alignment to a subtelomere reference as the first step of the pipeline. We hope that the inclusion of this data in our manuscript will be a note of caution for others.

- For the tests on clinical samples, B2_duplex and B2_simplex, initially there were 2,034 and 3,170 telomeric reads. Later, it was mentioned that the number of telomeric reads were increased to 6,266, which improved the results. It was not clear how the authors increased the number of telomeric reads to 6,266. Did they use ONT reads generated with higher coverage depths or something else?

The higher depth mentioned here relates to a different sequencing run of a different clinical sample. Unfortunately, this sample cannot be included in this paper due to the inability to make the underlying dataset publicly available at this point in time. We merely wanted to inform the readers that this approach works well on non-HG002 clinical samples when a sufficient number of reads are available. However, in light of the reviewer’s comment, the corresponding statement has been removed from the text.

- How did the authors conclude that a minimum of 5,000 telomeric reads are required per sample for de novo chromosome arm-specific telomere length analysis?

We noticed a pattern between increasing the number of telomeric reads and the efficiency of the clustering algorithm, however, the 5,000 telomeric reads is not supported by data and was therefore removed from the manuscript~~.~~

Minor Comments:

- There are a few typos and inconsistencies. Before publication, the manuscript must go through thorough proof-reading. For example:

-- Page 15, Line 352-353: “... SUP basecalling basecalling (Fig. 3c).” basecalling is written twice.
-- Page 20, Line 483: has an extra space here, “... identified .”
-- Figures 5b and 5f are missing “[” and “]” in their y-axis labels
-- BP and bp are used interchangeably; should be consistent
-- For referring to coverage, “X” and “x” are used interchangeably; should be consistent
-- Page 23, Line 548: should be “Supplemental Fig. 7a”
-- Page 31, Line 757: “... de novo telomeres reads …”; should be telomeric reads.

We thank the reviewer for their attention to detail. The corresponding changes have been made in the text.

-- Page 15, Line 363: “... after the identification of twenty telomeric repeats.” Previously, it was mentioned that ten telomeric repeats.

The parameter using ten telomeric repeats was used to isolate putative telomeric reads from the remainder of the sequencing data. At a later point in time, when identifying the capture probe, TARPON searches for the first capture probe after 20 telomeric repeats. The threshold at this stage was increased to 20 as the capture probe sequence with two errors used was found in one of the patient’s subtelomeric sequence. To decrease the computation time, 20 is not used during the initial identification of putative telomeres.

- It is not clear what the three plots in supplemental figure 2 are referring to. A better explanation is required for the ease of understanding.

These graphs depict reads that terminate in a capture probe but lack a region of invariant wild type telomeric repeats. The text has been updated for clarity. “Supplemental Figure 2. (a) Three examples of reads that end in a capture probe but lack a region of invariant telomeric repeats, instead terminating within the variant repeat rich region. Blue lines represent the frequency of GGTTAG repeats within a 100 bp sliding window, orange lines represent the frequency of all telomere+1N repeats within a 100 bp sliding window, and red lines represent the VRR-region start site.”

The corresponding part of the result section has been modified as follows (lines 315-322):

“The region enriched for the variants is referred to as the variant repeat–rich (VRR) region and varied in length from a few hundred base pairs (Fig. 4a, left) to several kilobases (Fig. 4a, middle and right) before transitioning into invariant GGTTAG repeats. As this telomere repeat containing region may arguably have functional relevance, we have included it in the telomere length output. This decision is supported by observations of reads that terminate in a capture probe specific to the enrichment methodology but essentially devoid of a terminal stretch of invariant GGTTAG repeats as seen in Fig. 4a (Supplemental Fig. 2a, blue line).”

- The authors should update the link to the software’s GitHub repository since the one mentioned in the manuscript is not being maintained any more.

The manuscript and the details composed therein are referencing the version 1.0 of TARPON available at the GitHub repository referenced in the manuscript. This version is stable with no known issues and its default parameters match those found within this manuscript. The GitHub provides an additional link to a version of TARPON that contains additional features presently under development and subject of a later publication.

- I tried to install TARPON in my macbook locally and execute the pipeline on the provided sample dataset following the instructions from the GitHub repository:

-- First, it failed to execute as the default # of CPUs were set to 10, but my machine has 8 cores. Can the source code be modified to automatically take the maximum number of CPUs available in the machine it is running on, if it is less than 10?

This issue does not arise with TARPON 1.0 described in this manuscript but is specific to the development version linked on our GitHub. Thank you for bringing this issue to our attention. Older versions of TARPON, prior to the implementation of the –threads parameter were tested on wsl, Linux, and macOS machines however, this was not done after setting the number of default threads to ten. The source code has been modified to use the threads value when possible, or if this value exceeds the maximum number of threads available, to utilize the appropriate number of cpus found on the device. To prevent similar issues for readers of this manuscript, the reference to the development version of TARPON has been updated on GitHub.

-- Next, I executed adding the “--threads” parameter and setting its value to 8. This time, it exited on another error related to the docker. I assume the software is not locally executable on macOS. If that is the case, it should be mentioned in the manuscript that the stand-alone version is platform-dependent.

The software is locally executable on macOS and has been recently tested on macOS Ventura 13.0, Monday October 27^th^, 2025. The stand-alone version is not platform-dependent and has been tested on macOS, WSL, and Linux based machines. If the reviewer is continuing to have problems the authors invite the reviewer to open an issue on the GitHub repository and provide a description of the error so that it can be better addressed. Without the error message, it is difficult to hypothesize what the issue may be, however the authors believe it may be an issue with the reviewers docker desktop application not being open or the user not being added to the docker group upon the first execution of TARPON when it attempts to download the appropriate docker image. MacOS interface with docker is slightly more complicated than that of Linux or WSL systems. We would greatly appreciate hearing more of this issue as it may be something other uses face in the future.

**Reviewer #3**

In this manuscript, authors intended to show TARPON is a comprehensive and modular pipeline for Nanopore-based telomere analysis, offering high flexibility. While the default settings are optimized for telomerase-positive human samples, all parameters are easily adjustable via the GUI or command line. This flexibility allows TARPON to support the analysis of organisms with non-canonical telomeric repeats and variant repeat-rich samples.

However, TARPON clearly acknowledges its limitations.
1. Limitation for Heterogeneous Repeat Structures: TARPON is currently not recommended for organisms with highly heterogeneous sequence repeats. This is an honest admission that TARPON's core VRR definition methodology is designed to handle relatively uniform Telo+1N variations and is not suitable for complex, heterogeneous repeat structures.
2. Clinical Validation Status: The manuscript explicitly states that neither Nanopore sequencing technology nor the TARPON pipeline has been formally clinically validated and should not be used as the sole clinical diagnostic methodology at this time. This disclaimer is important for maintaining scientific rigor and promoting responsible technology usage.
These limitations clearly define TARPON's current scope and emphasize the need for further research towards clinical adoption.

 We appreciate the reviewer’s positive evaluation and thoughtful summary of TARPON, along with their valuable critiques. In the following responses, we have addressed their recommendations.

There are some recommendations to be addressed for the improvement of the manuscript

1. Although elaboration on the comparison among different existing telomere analysis methods can be found, a formal figure seems to be necessary to address the differences in actual data output to provide more information regarding the benchmarking process.

The programs are sufficiently different to impair a simple comparison for bench-marking purposes. Wf-teloseq and TARPON both aim to provide a full analysis pipeline from basecalled sequencing data to output. However, wf-teloseq is only capable of handling C-strand telomeric reads, not G-strand reads. In contrast, Telometer and TeloBP require pre-execution filtering and capture probe identification and passing raw telomeric sequences would not be appropriate use of these programs. We therefore used 400 manually annotated telomeric sequences and modified each of the programs above to calculate the length of the telomeres within (lines 630-640, “Since each software utilizes a slightly different approach than TARPON to identify the subtelomere-to-telomere boundary, the 400 manually curated telomeric sequences were used to calculate the mean absolute value error of wf-teloseq as 157 nucleotides, of Telometer as 161 nucleotides, and of TeloBP as 29.8 nucleotides, compared to the 4.03 nucleotide mean absolute value error of TARPON (Supplemental Fig. 7b). Additionally, six reads within Telometer and 10 reads withing wf-teloseq had an absolute value error greater than 500 nucleotides with a maximum error of 10,140 nucleotides in wf-teloseq driven primarily by the identification of telomere islands within the subtelomeric sequences (Supplemental Fig. 7c). While these telomere islands may contribute to chromosome stability and exhibit shelterin binding, the intervening nontelomeric sequence most likely does not and would result in a non-biological chromosome-arm specific telomere length bias.”)

2. The wf-teloseq MAE of 42nt must be removed. Instead, TARPON (4.36nt) should be compared to wf-teloseq's raw MAE (157nt), and the reasons for wf-teloseq's susceptibility to misidentifying subtelomeric islands (algorithmic flaw) should be described in a separate paragraph. The post-filtered comparison result is not statistically justifiable.

The wf-teloseq MAE of 42 nt has been removed and TARPON (4.36 nt) is now compared to wf-teloseq's raw MAE (157nt).

3. The manuscript must explicitly acknowledge that including the VRR region in telomere length measurement causes a systematic length difference (overestimation) compared to traditional canonical repeat-based measurement methods, and quantitative data on this difference should be presented to assist readers in interpreting the measurement results.

A supplementary figure has now been added to show the difference in calculated telomere length when comparing from the start of the VRR region to the end of the telomere and the number of nucleotides composed of wild type telomeric repeats in the same stretch of sequence. (lines 401-407, “The VRR-region is composed primarily of telomeric and telomere+1N repeats. However, including this region in the calculation of telomere length will result in an increased telomere length when compared to techniques that determine the telomere start solely based on the frequency of invariant telomeric repeats. This difference depends highly on the sequence structure of the VRR-region. There is a mean telomere length difference of 552±273 bases when comparing the length of the VRR-region containing telomere to the summation of all wild type telomeric repeats within the same region (Supplemental Fig. 4b)”).

4. The more accurate term "Telomere Allele Specific Telomere Length" must be consistently used throughout the manuscript instead of "Chromosome Arm-Specific Telomere Length" when describing de novo clustering results.

This has been updated in all relevant locations to easily distinguish between chromosome arm specific telomere lengths (when performing alignment) and allele specific telomere lengths (when performing clustering).

5. The high proximal truncation read removal rate (∼50%) observed in Duplex-enriched libraries (especially HEK-DE) should be highlighted as an experimental limitation of the Duplex Capture protocol. Users should be warned that this method may yield a lower proportion of usable full-length reads compared to the Splint-enriched method.

The results have now been updated to emphasize the reduced number of G strand telomeric sequences that do not contain a capture probe; however, in our experience the number of telomeric sequences arising from the Duplex Enrichment approach is much greater than that of the simplex approach. This data was not presented within the manuscript as telomere enrichment techniques are constantly evolving and improving and the wet lab methodologies to enrich for telomeres is not the main focus of this manuscript. (lines 695-705, “Here, the authors presented two simplex enrichment and two duplex enrichment sequencing experiments. While a large fraction of G strand telomeric reads in the duplex enrichment methodology do not contain a capture probe and are therefore removed from the analysis, no notable differences in final telomere read count were apparent and read count differences are more likely a consequence of flowcell health than enrichment protocol differences. However, differences between DNA extraction methods greatly impacts the number of telomeric reads sequenced with SE_HG002 and DE_HEK containing 8,286 and 7,520 telomeric reads respectively, while the robotically extracted, lower molecular weight DNA of SE_WB60 and DE_WB60 results in 3,171 and 2,034 telomeric sequences respectively. Further analysis to understand the influence of DNA extraction quality on telomere enrichment will be necessary to ensure maximal protocol efficacy.”).

6. Final confirmation should be made that the putative telomere sequence UBAM files for the four samples used in the study will be made publicly available on SRA at the time of manuscript publication, as stated in the Data Availability Statement. Adherence to this commitment is crucial for ensuring scientific transparency.

The authors are excited to report that the four sequencing files are now publicly available under SRA and the manuscript has been appropriately updated to reflect this (line 803,“Putative telomeric sequences containing at least ten non-consecutive telomeric repeats are provided for the four samples described in this study as supplemental data files in UBAM format and will be publicly available on SRA under BioProject #PRJNA1313423”).

7. It is unclear about which basecaller method (Fast/SUP/hybruid format, etc.) was used in the later part of the analysis (corresponding to the results in Fig.3 and on) inside the manuscript. It would be great to include such information inside the manuscript.

SUP basecalling was used for Fig 3. and onwards. The text has now been updated to reflect this (line 245, “Therefore, unless stated otherwise, all results originate from SUP basecalled reads.”).

8. A figure representing the TARPON method (+ other methods like TeloBP / described in the discussion section of the manuscript) regarding identification of the telomeric/subtelomeric region boundary would be substantially beneficial (since this is critical to not only define the telomere sequence, but also the telomere length, which is imperative in cancer research area).

A figure illustrating the differences between the available software pipelines and their respective capabilities has been added as supplemental figure 8. The methods used to define the subtelomere/telomere boundaries are described in the discussion and are sufficiently different to make graphical representation exceedingly difficult for us. We also think that evaluation of these different methodologies must involve experiments that directly address which method best approximates functional telomere length. Such analysis is best left to a future biochemical study.
